# Importance of naturalized breeds as a base for the formation of exotic sheep (*Ovis aries*) breeds in tropical altitude regions

**DOI:** 10.1590/1678-4685-GMB-2019-0054

**Published:** 2020-05-29

**Authors:** Joyce Azambuja de Oliveira, Andrea Alves do Egito, Bruno do Amaral Crispim, Fernando Miranda de Vargas, Leonardo de Oliveira Seno, Alexeia Barufatti

**Affiliations:** 1 Universidade Federal de Grande Dourados, Faculdade de Ciências Biológicas e Ambientais, Dourados, MS, Brazil.; 2 Embrapa Gado de Corte, Campo Grande, MS, Brazil.; 3 Universidade Federal de Grande Dourados, Faculdade de Agráras, Dourados, MS, Brazil.

**Keywords:** Ovis aries, sequencing, Pantaneira breed, maternal inheritance, mitochondrial DNA

## Abstract

Recent molecular tools and technologies have marked the discovery of the origin and domestication processes of a wide variety of species, using either genomic or mitochondrial molecular markers to provide input for selection programs, as well as the management and conservation of animal breeds. This study aimed to analyze genes of mitochondrial DNA of the following sheep (*Ovis aries*) breeds: Pantaneira, Bergamácia, Dorper, White Dorper, Ile de France and Hampshire Down, to obtain their population genetic parameters and investigate the origin of these sheep populations reared in the State of Mato Grosso do Sul. The analysis of mitochondrial DNA allowed to infer their phylogenetic relationships and revealed significant differences among them when compared with each other and with sequences obtained from GenBank. Through the formation of haplotypes, it was noted that the Pantaneira breed served as the maternal basis for the formation of the other breeds reared in the region, and it was possible to suggest a European origin for the sheep populations studied.

## Introduction

Brazil has several breeds of sheep (*Ovis aries*), including those that were developed from breeds imported by settlers soon after their discovery. Over the years, these animals underwent natural selection, resulting in breeds that are now considered naturalized, locally adapted, or native (Mariante *et al.*, 1999).

Conservation programs using molecular tools are crucial for the generation of information about the genetic diversity of locally adapted groups, thus allowing them to be included in production systems for integration of adaptation and rusticity features (Barker, 1994). Knowledge of the population structure combined with information about genetic changes can influence future management actions and can be used to develop strategies for using a breed in a particular ecosystem as a model for genetic improvement programs (Paiva *et al.*, 2011). The importance of locally adapted livestock breeds is becoming recognized through their contribution to food security in marginal land areas of the world. Reviews by the FAO and other international agencies highlight the crucial significance of these breeds, both as sustainable resources of food, but also as living reservoirs of biodiversity, providing genetic adaptive fitness traits to improve mainstream agriculture (FAO, 2009; Hoffmann, 2013).

Molecular tools and technologies have marked the discovery of the origin and domestication processes of a wide variety of species by means of genomic or mitochondrial molecular markers. These tools have aided in the understanding of evolutionary relationships, taxonomy, and demography of several species that can provide support to identify the most important areas for conservation programs, in addition to the analysis of genetic diversity in domestic, wildlife, and endangered species (Rosa and Paiva, 2009; Grisolia and Moreno-Cotulio, 2012).

The Pantaneira breed presents a higher genetic variability than other breeds of sheep reared in tropical altitude regions. Therefore, the development of research that aims at their conservation and genetic improvement is important (Crispim *et al.*, 2013).

The study of locally adapted breeds by menas of mitochondrial markers allows to recognize the origin and to genetically characterize the different populations that possibly comprise them. Several studies were made to determine the phylogenetic origin of criollo or endemic breeds from a particular country (Bravo *et al.*, 2015; Liu *et al.*, 2016; Resende *et al.*, 2016; Ghernouti *et al.*, 2017; Moradi *et al.*, 2017). Therefore, this study aimed to evaluate different genes of mitochondrial DNA in naturalized and exotic breeds of sheep in tropical altitude regions to obtain population genetic parameters and investigate the origin of these Brazilian populations.

## Material and Methods

### Samples and sequencing

Blood samples from 40 animals from six sheep (*Ovis aries*) breeds (Pantaneira, Bergamacia, Dorper, White Dorper, Ile de France and Hampshire Down) reared in the central west tropical altitude region in the State of Mato Grosso do Sul (MS), Brazil, were collected and used for DNA extraction and sequencing (Table 1). The Pantaneira and Bergamacia breeds were considered as naturalized, whereas the exotic breeds included Dorper, White Dorper, Ile de France, and Hampshire Down. DNA extraction was performed using a whole blood DNA extraction protocol described by Crispim *et al.* (2012). Additionally five sequences deposited in GenBank representing each of the five existing mitochondrial ovine haplogroups (A, B, C, D and E) were used in this study. They were deposited under the accession numbers KF302446 (Merinizzata Italian breed), KF302447.1 (Lacaune breed) (Lancioni *et al.*, 2013), HM236178 (Karakas breed), HM236180 (Morkaraman breed) and HM236182 (Awassi breed) (Meadows *et al.*, 2011), respectively.

**Table 1:** Breeds, number of individuals (N), site of collection of the animals in the State of Mato Grosso do Sul (MS), number of haplotypes (NH), haplotype (H), and nucleotide (π) diversity.

Breeds	Abbrev.	N	Site	NH*	H	π
Pantaneira	PT	15	Dourados/Corumbá	7	0.8571 ± 0.0645	0.0024 ± 0.0014
Bergamácia	BE	5	Jardim	3	0.7000 ± 0.2184	0.0023 ± 0.0017
Ile de France	IF	6	Campo Grande	5	0.9333 ± 0.1217	0.0038 ± 0.0024
Dorper	DP	5	Caarapó	4	0.9000 ± 0.1610	0.0011 ± 0.0009
White Dorper	WD	4	Caarapó	4	1.0000 ± 0.1768	0.0038 ± 0.0028
Hampshire Down	HS	5	Ponta Porã	3	0.7000 ± 0.2184	0.0022 ± 0.0016
**TOTAL**		**40**		19		

* Each breed had a specific number of haplotypes, but many breeds share the same haplotype. Therefore the sum of the haplotypes per breed is greater than the total number found in this study.

Fragments from three mtDNA loci: NADH dehydrogenase (ND5), cytochrome b gene (CytB) and control region (D-loop), were amplified (Table 2). The amplified fragments were purified and sequenced using the Sanger sequencing technique (Sanger *et al.*, 1977) in an ABI 3730 XL automatic sequencer (Applied Biosystems).

**Table 2:** Primers used in amplification reactions. Shown are size of the fragments (SF) and annealing temperature (AT) for each locus.

Primers	Primer Sequence (5’- 3’)	SF (bp)	AT (°C)
CytB F^1^	ACCTCCTTTCAGCAATTCCA	765	60 °C
CytB R^1^	AGGGAGGTTGGTTGTTCTCC		
ND5 F^2^	AATAGTTTATCCAGTTGGTCTTAGG	657	52 °C
ND5 R^2^	AAGATTTGTTGGAGATCTCAGGTG		
D-loop F^*^	ACAAACCCACATAACAACCC	716	60 °C
D-loop R^*^	GGCTGATTAGTCATTAGTCCA		

* Designed using the PerlPrimer software from the sequence AF010406 (Hiendleder *et al.*, 1998).

### Data analysis

The sequences were edited and aligned using DNA Alignment software (Fluxus Technology Ltd, www.fluxus-engineering.com) with the reference sequence AF010406 (Hiendleder *et al.*, 1998). The analysis of molecular variance (AMOVA), Wright’s F-statistics (F_ST_), haplotype (H) and nucleotide (π) diversity were all calculated in ARLEQUIN 3.5 software (Excoffier and Lischer, 2010). The relationship between the generated haplotypes was estimated by constructing haplotype networks with Network software version 4.1.1.2 (Fluxus Technology Ltd.) with the Median-Joining method (Bandelt *et al.*, 1999). For the analysis, the animals were divided into three different groups: 1 - each MS population individually; 2 - naturalized breeds *vs.* exotic breeds; 3 - MS populations *vs.* representatives of GenBank reference sequences.

## Results

### Haplotype analysis

Forty mtDNA sequences of 1350 bp were analyzed in this study, and 19 haplotypes were found in the populations. None of these haplotypes were similar to those deposited in GenBank, representing the five existing haplogroups (A, B, C, D and E) identified by Wood and Phua (1996) and classified by Hiendleder *et al.* (1998). Therefore, when these samples were included in the analysis, 24 haplotypes were found for the 45 sequences.

Data related to haplotype (H) and nucleotide (π) diversity for each studied breed, based on the analysis of different genes of mitochondrial DNA, are described in Table 1. The White Dorper and Ile de France breed presented the highest values of haplotype and nucleotide diversity while the Pantaneira breed presented seven of the 19 found haplotypes.

### Analysis of molecular variance

The results of AMOVA for the three groups, analyzed using different genes of mitochondrial DNA, are shown in Table 3. When AMOVA was done comparing each MS population individually and naturalized breeds *vs.* exotic breeds, the molecular variance within populations was higher than among populations, as expected. However, when AMOVA was performed comparing MS populations *vs.* representatives of GenBank reference sequences, the percentage of variation was inverse, that is, higher between populations (> 60%) than between individuals (> 30%).

**Table 3:** Analysis of molecular variance (AMOVA) of the populations of the State of Mato Grosso do Sul (MS) examined with different genes of mitochondrial DNA.

Groups^a^	% of variation (DF)^b^		F_ST_
	Among populations	Within the populations	
1	8.46% (5)	91.54% (34)	0.08
2	4.06% (1)	95.94% (38)	0.04
3	66.22% (10)	33.78% (34)	0.66

It was possible to observe a genetic differentiation between the different breeds raised in MS (*p* < 0.05), but the differentiation was not observed when the comparison was done between naturalized and exotic breeds. Higher values of F_st_ (0,66) were observed when the Brazilian breeds were compared with the five exotic GenBank sequences that represented the distinct haplogroups.

### Population structure

**Figure 1:**
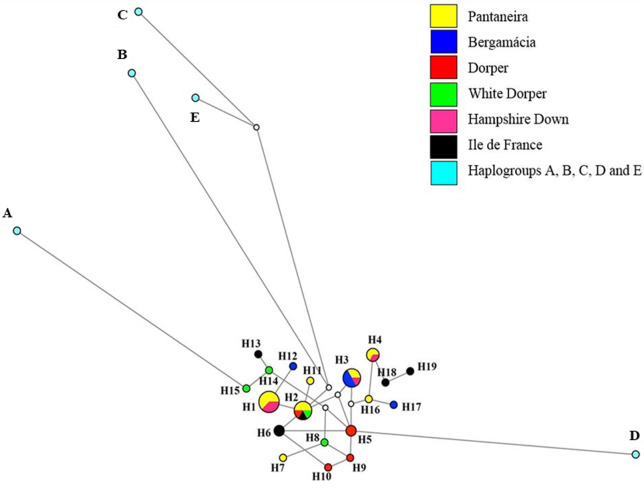
Network formed by the Median-Joining method, demonstrating the 24 haplotypes found for the different mtDNA genes in the GenBank reference sequences (A, B, C, D and E) and the State of Mato Grosso do Sul populations. The areas of the haplotype circles are proportional to their frequency. Line length is related to mutational steps separating each haplotype. The white dots are mean vectors representing hypothetical haplotypes introduced by the algorithm.

**Figure 2:**
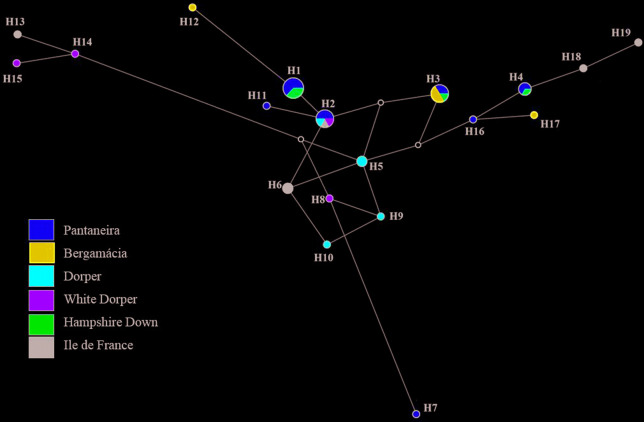
Median-joining network showing the 19 haplotypes found with different mtDNA genes for populations of the State of Mato Grosso do Sul. The areas of the haplotype circles are proportional to their frequency. Line length is related to mutational steps separating each haplotype. The white dots are mean vectors representing hypothetical haplotypes introduced by the algorithm.

Figures 1 and 2 show the haplotype networks constructed for the populations of MS, individually and together with the sequences from GenBank, based on point mutations in the sequences, demonstrating the relationship between the different haplotypes formed. The closest haplogroups of the Brazilian breeds were A and D, but by the network formed it was not possible to know to which of them our populations belong to (Figure 1). There was a higher frequency of H1, H2, H3, and H4 haplotypes in more than one breed. In contrast, in the sample evaluated, all breeds had unique haplotypes, except for Hampshide Down (Figure 2).

## Discussion

### Haplotype analysis

Variability in mtDNA was found in populations of the State of Mato Grosso do Sul (MS), as individuals were distributed in 19 haplotypes (Table 1). Each White Dorper individual had a distinct haplotype, therefore this breed presented the highest value of haplotype diversity. The Pantaneira breed presented seven of the 19 haplotypes found, which could be atributed to their ancient presence in the state, supported by the history of introduction of these breeds in MS. In a study carried out with microsatellite markers, Crispim *et al.* (2013) also observed high genetic variability in the Pantaneira breed animals when compared to the others.

### Analysis of molecular variance

Hartl and Clark (2010) suggested the following guidelines for the interpretation of F_ST_: 0-0.05 (negligible), 0.05-0.15 (moderate), 0.15-0.25 (significant), and above 0.25 (high genetic differentiation). According to these authors, the F_ST_ value for Group 1, where only the populations of the State of Mato Grosso do Sul were analyzed, indicated a moderate genetic differentiation (0.08). In Group 2 (naturalized breeds *vs*. exotic breeds) there was slight genetic differentiation between animals (0.04). In Group 3, where the GenBank reference sequences were included, the F_ST_ value was 0.66 indicating high genetic differentiation. In group 3, the high observed variation between the populations evaluated in this study and the populations represented in GenBank may have been generated by the absence of haplotypes that represent the haplogroups described in the ovine species in the MS breeds. In groups where genetic differentiation was moderate (Group 1) and high (Group 3) there was also a significant genetic distance between the individuals (Table 3).

White Dorper animals originated in South Africa from interbreeding, where one parent was of Asian origin (ARCO, 2011), thus elucidating the genetic distance observed when compared to the Pantaneira breed. The Brazilian Bergamácia breed is the result of processes of adaptation and natural selection of animals imported into Brazil, with the last import occurring during the 1930s. Since then, the animals of the Brazilian Bergamácia breed have been isolated from their Italian ancestors and, although they have been on Brazilian land for several years, the breed standard was created only in 1977 (ARCO, 2013). Dorper is an exotic breed, also originating from South Africa, with maternal heritage of Asian origin in its mitochondrial genome, which may be the reason for the distance found.

### Population structure

With the exception of the Hampshire Down breed, all breeds evaluated have different haplotypes. In the most frequent haplotypes (Figure 2), it can be observed that the Pantaneira breed has haplotypes shared with the exotic breeds. This evidences that the Pantaneira breed is being used as a maternal lineage for the formation of exotic breed herds. This fact was also evidenced by Crispim *et al.* (2013), who, using microsatellite markers, observed an allelic sharing between these breeds.

When comparing the Pantaneira breed with animals of the Creole breed of Southern Brazil, using the ND5 gene of mitochondrial DNA, Oliveira *et al.* (2015) found significant differences between the two breeds, suggesting the occurrence of differentiation. Analysis of mtDNA variation, using mainly control regions (D-loop), cytochrome b, and cytochrome oxidase I, revealed the existence of five haplogroups (A, B, C, D, and E) in domestic sheep (*Ovis aries*) sampled from various geographically disperse regions. Among these five, A and B are the haplogroups identified with greater frequency, and they grouped with the animals of Asian and European origin, respectively. Both were first identified by Wood and Phua (1996) and then classified by Hiendleder *et al.* (1998); however, they have been reported in all geographic regions where *Ovis aries* was sampled.

Haplotypes H14 and H15 (Figure 1), formed by animals of the White Dorper breed, were closer to the GenBank reference sequence representing the haplogroup A (Asian origin), thus indicating the possibility of this breed having Asian maternal inheritance in its mitochondrial genome. This possible Asian heritage may be justified by the recent import of embryos of this breed. The remaining haplotypes formed were closer with haplogroup B, albeit owing to the mean vectors representing hypothetical haplotypes, indicating the possible European origin of these populations.

The analysis of mitochondrial DNA provided an overview of phylogenetic relationships among populations of sheep in the State of Mato Grosso do Sul, as well as significant differences between them when compared with each other and with the sequences obtained from GenBank.

Through the formation of the haplotypes, it was observed that the Pantaneira breed served as the maternal basis for the formation of other breeds reared in the region. This puts in evidence the urgent need to maintain conservation nuclei of the Pantaneira breed to safeguard the genetic basis of this population, avoiding that it can be used indiscriminately in absorbent crosses. Although it was possible to suggest a European origin for the sheep populations studied, further studies using more representative sequences are needed to determine their origins.[Bibr B25] [Bibr B26]
